# Adverse Impact of Heavy Metals on Bone Cells and Bone Metabolism Dependently and Independently through Anemia

**DOI:** 10.1002/advs.202000383

**Published:** 2020-08-04

**Authors:** Shuping Zhang, Li Sun, Jie Zhang, Sijin Liu, Jinxiang Han, Yajun Liu

**Affiliations:** ^1^ The First Affiliated Hospital of Shandong First Medical University Jinan Shandong 250014 China; ^2^ Biomedical Sciences College & Shandong Medicinal Biotechnology Centre Shandong First Medical University & Shandong Academy of Medical Sciences Jinan Shandong 250062 China; ^3^ State Key Laboratory of Environmental Chemistry and Ecotoxicology Research Center for Eco‐Environmental Sciences Chinese Academy of Sciences Beijing 100085 China; ^4^ Beijing Jishuitan Hospital Peking University Health Science Center Beijing 100035 China

**Keywords:** heavy metals, degenerative bone diseases, anemia, adverse outcome pathways, mode of actions

## Abstract

Mounting evidence is revealing that heavy metals can incur disordered bone homeostasis, leading to the development of degenerative bone diseases, including osteoporosis, osteoarthritis, degenerative disk disease, and osteomalacia. Meanwhile, heavy metal‐induced anemia has been found to be intertwined with degenerative bone diseases. However, the relationship and interplay among these adverse outcomes remain elusive. Thus, it is of importance to shed light on the modes of action (MOAs) and adverse outcome pathways (AOPs) responsible for degenerative bone diseases and anemia under exposure to heavy metals. In the current Review, the epidemiological and experimental findings are recapitulated to interrogate the contributions of heavy metals to degenerative bone disease development which may be attributable dependently and independently to anemia. A few likely mechanisms are postulated for anemia‐independent degenerative bone diseases, including dysregulated osteogenesis and osteoblastogenesis, imbalanced bone formation and resorption, and disturbed homeostasis of essential trace elements. By contrast, remodeled bone microarchitecture, inhibited erythropoietin production, and disordered iron homeostasis are speculated to account for anemia‐associated degenerative bone disorders upon heavy metal exposure. Together, this Review aims to elaborate available literature to fill in the knowledge gaps in understanding the detrimental effects of heavy metals on bone cells and bone homeostasis through different perspectives.

## Graphical abstract

Exposure to heavy metals can induce degenerative bone diseases, including osteoporosis, osteoarthritis, and degenerative disk disease, through anemia‐independent or likely through anemia‐independent routes. In the current Review, the underlying modes of action (MOAs) and adverse outcome pathways (AOPs) responsible for each route are recapitulated. The MOAs underlying anemia‐independent routes mainly include altered osteogenesis and osteoblastogenesis, imbalanced bone formation and resorption, disturbed Ca/P homeostasis, and inhibited mineralization. On the other hand, stress erythropoiesis‐driven remodeling of bone microarchitecture, renal erythropoietin‐hypoproduction, and disordered iron homeostasis are involved in anemia‐dependent routes. Furthermore, oxidative stress is a common mechanism involved in both anemia‐dependent and ‐independent routes.

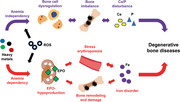

## 1 Introduction

**Figure 1:**
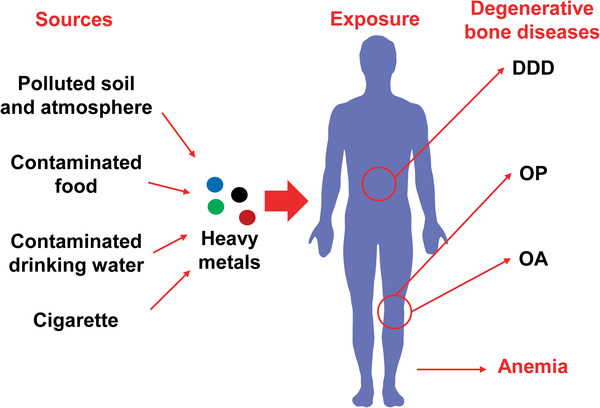
Degenerative bone diseases and anemia under exposure to heavy metals. Polluted soil and atmosphere, contaminated food, drinking water, and cigarette smoke are the main sources for heavy metal exposure. Mounting evidence reveals that heavy metal exposure increases the risk of anemia and the development of degenerative bone diseases, e.g., osteoporosis (OP), osteoarthritis (OA) and degenerative disk disease (DDD).

Heavy metals are dense metals with a high atomic number and atomic weight with a specific gravity greater than 5, and most are usually toxic even at low doses. Heavy metals comprise basic metals, transition metals, metalloids, actinides and lanthanides. Typical heavy metals refer to cadmium (Cd), lead (Pb), mercury (Hg), and chromium (Cr). However, iron (Fe), copper (Cu), aluminum (Al), zinc (Zn), beryllium (Be), cobalt (Co), manganese (Mn), and arsenic (As) may also be deemed as heavy metals dependent on the context. Notably, while beneficial heavy metals exhibit adverse outcomes only at high levels, detrimental ones can be toxic even at trace levels.^[^ ^1^ ^]^ Heavy metals (referring to detrimental metals in the current review) are mainly incorporated into the human body via the consumption of contaminated food and drinking water, as well as respiration (e.g., cigarette smoke) (**Figure** **1** and Table S1, Supporting Information). Particularly, cigarettes are composed of hundreds of ingredients, including nicotine and heavy metals. In tobacco filled in cigarettes, the range of heavy metal concentrations varies largely, depending on the brand and origin of cigarettes.^[^ ^2^, ^3^ ^]^ As it is difficult to excrete heavy metals, they can be retained, accumulating in vital organs and tissues, including the liver, kidney, heart, and brain, for several years or even decades.^[^ ^4^ ^]^ Exposure to heavy metals has been recognized as one of the main threats to human health globally, inducing a variety of detrimental outcomes or even diseases, including neuronal toxicity, anemia, cardiovascular disorders, renal injury, diabetes, and cancers.^[^ ^5^ ^]^ For example, as typical heavy metals, Cd and Pb can damage the kidney, liver and lung, and induce anemia, nephrotoxicity, heart failure, and diabetes.^[^ ^6^, ^7^, ^8^ ^]^ Heavy metals can induce detrimental effects on humans through various mechanisms, such as oxidative stress,^[^ ^9^, ^10^ ^]^ even exerting synergistic effects with other pollutants.^[^ ^11^ ^]^

Furthermore, mounting evidence revealed through epidemiological, in vivo, and in vitro studies that heavy metals can induce disordered bone homeostasis and are involved in the development of degenerative bone diseases, including osteoporosis (OP), osteoarthritis (OA), degenerative disk disease (DDD), and osteomalacia, particularly OP (Figure 1). Itai‐itai disease is the first and most well‐known Cd‐induced disease, characterized by proteinuria, osteomalacia, OP, and pain in the back and legs resulting from kidney damage. Along with the extension of the femoral and back pain to the rest of the body, patients are finally bed‐ridden owing to bone fractures.^[^ ^12^, ^13^ ^]^

OP is a widely prevalent disease, characterized by impaired bone mass and microarchitecture, resulting in fragility fractures. It is largely induced owing to disrupted orchestration between bone‐forming osteoblasts and bone‐resorbing osteoclasts through diverse mechanisms.^[^ ^14^ ^]^ As the most common type of joint disease, OA plays a major role in inducing pain and disability worldwide, affecting 10% of men and 18% of women over the age of 60 years. However, the etiology and pathogenesis of OA are complicated by the involvement of pathological changes in the cartilage, subchondral bone and synovium.^[^ ^15^ ^]^ DDD is an age‐related pathological condition occurring when one or more discs between the vertebrates weakens, resulting in pain.^[^ ^16^ ^]^

Anemia is mainly characterized by a decrease of circulating red blood cells (RBCs) and can be induced via various etiologies, including iron deficiency (ID), infections, cancers, chronic kidney disease, and genetic disorders. Typically, anemia is attributed to direct damages to circulating RBCs and impaired erythropoiesis, a process involving the differentiation and maturation of erythroblasts.^[^ ^17^ ^]^ Currently, anemia affects about one‐third of the global population and remains a major cause of morbidity and mortality worldwide.^[^ ^17^, ^18^ ^]^ Common types of anemia include iron deficiency anemia (IDA), anemia of inflammation, sickle cell disease (SCD) and thalassemia.^[^ ^17^, ^18^ ^]^

Exogenous etiologies have emerged as notable factors in the pathogenesis of both degenerative bone diseases and anemia. Growing evidence has revealed that the uptake and accumulation of heavy metals is one such etiological factor. In addition to occupational exposure, heavy metals frequently accumulate in elderly individuals who present a high risk for both degenerative bone diseases and anemia. Furthermore, individuals with anemia have a higher risk of developing OP and OA. Nonetheless, the cause‐effect relationships and the interplay among heavy metals and these adverse outcomes are not well‐understood and not well‐recapitulated. A systemic review regarding their relationships is necessary and imperative to raise concerns about heavy metal‐induced adverse outcomes. In the current review, we recapitulate the contributions of heavy metals to the incurrence of degenerative bone diseases dependently and independently on anemia. Furthermore, we shed light on the underlying modes of action (MOAs) and adverse outcome pathways (AOPs) responsible for degenerative bone diseases and anemia under exposure to heavy metals. Additionally, we highlight the potential role of heavy metals in disrupting the orchestration of erythropoiesis, iron homeostasis and bone hemostasis. An improved and systemic understanding of how heavy metals induce degenerative bone diseases will help elucidate their adverse effects on human health and develop corresponding strategies for the prevention and treatment in high risk populations, such as the elderly and cohorts occupationally exposed to heavy metals. The primary purpose of this review is to update knowledge and identify gaps in understanding the contribution of heavy metals to the development of degenerative bone diseases, appealing to a broad interest, and to promote more research efforts in this field.

## 2 Epidemiological Evidence of the Occurrence of Heavy Metal‐Induced Degenerative Bone Diseases and Anemia

### 2.1 Heavy Metal‐Related Degenerative Bone Diseases

Cd is the best‐characterized heavy metal involved in bone homeostasis disorder and OP. A cohort study, including 820 postmenopausal women (aged 53–64 years), reported that the bone mineral density (BMD), parathyroid hormone and urinary deoxypyridinoline (U‐DPD) values negatively correlated with Cd levels in the urine. A significant interaction between Cd and menopause was observed for U‐DPD.^[^ ^19^ ^]^ Another cohort study, including 2676 postmenopausal women (aged 56–69 years), also demonstrated that higher Cd levels were associated with BMD in the total body and lumbar spine. Following a high dietary cadmium exposure, a 32% increased risk of OP and a 31% increase in the risk for the first fracture were observed when compared with lower exposures. Moreover, the odds ratios among never‐smokers were 2.65 and 3.05 for osteoporosis and fractures, respectively, after combining high dietary with high urinary cadmium.^[^ ^20^ ^]^ Similarly, Sadeghi et al. observed that the levels of Cd and Pb were 2.91 ± 0.18 ng mL^−1^ and 168.42 ± 9.61 ng L^−1^ in control women (*T*‐score ≥ −1), 2.97 ± 0.21 ng mL^−1^ and 176.13 ± 8.64 ng L^−1^ in woman patients with OP. For the woman patients with OP, the levels of Cd and Pb were 2.99 ± 0.1 ng mL^−1^ and 176.43 ± 13.2 ng L^−1^ in mild patients (−1 > *T*‐score > −1.7), and 3.80 ± 0.70 ng mL^−1^ and 221.44 ± 20 ng L^−1^ in severe patient group (*T*‐score < −1.7), respectively.^[^ ^21^ ^]^ In women, kidney biopsies indicated that Cd levels were positively associated with calcium (Ca) levels but were negatively associated with BMD.^[^ ^22^ ^]^ In a cohort that ingested Cd‐contaminated rice, a positive and significant correlation was observed between BMD changes and urinary and serum Cd levels in women (*p* < 0.01) but not in men (*p* > 0.05). A *Z*‐score analysis revealed that the prevalence of OP increased with increasing urinary and serum Cd levels in both women and men, especially in women with higher urinary and serum Cd levels.^[^ ^23^ ^]^ In smoking cohort, it was observed that Cd levels, as well as osteoprotegerin (OPG) and Ca levels, in the serum and urine were significantly increased in smokers occupationally exposed to residual Cd in plants when compared with non‐exposed smokers. More importantly, the occurrence of bony aches and joint pain in exposed smokers was significantly higher than that in non‐exposed smokers, indicating the effects of chronic Cd exposure on bone loss.^[^ ^24^ ^]^ Furthermore, Pb has been reported to be associated with disordered bone homeostasis. Women with Pb accumulation in the skeleton demonstrated thinner cortices in distal tibias and integral volumetric BMD. Moreover, a higher blood‐to‐bone Pb partition coefficient (PBB, log ratio) was associated with larger trabecular separation, lower trabecular volumetric BMD and trabecular number.^[^ ^25^ ^]^ Long‐term Pb exposure impaired osteoblastogenesis.^[^ ^26^ ^]^ Nonetheless, studies investigating the adverse effects of Pb on OP are still limited.

To date, data regarding the correlation between heavy metal exposure and OA remain limited. Cigarette smoke was reported to promote the development of OA.^[^ ^27^ ^]^ By evaluating Zn and Cd levels in the hip joint, it was concluded that a clear negative relationship existed between Zn and Cd levels in the articular tissue of OA patients.^[^ ^27^ ^]^ Additionally, high Cd levels were observed in the hip joints of patients with degenerative changes.^[^ ^28^ ^]^ Compared with the nonsmokers, cigarette‐derived Cd can contribute to a higher risk of cartilage loss and a more severe OA development.^[^ ^29^ ^]^

Moreover, Cd levels were negatively correlated with Zn levels in the intervertebral discs of patients with degenerative changes.^[^ ^30^ ^]^ In the intervertebral disc of patients presenting degenerative changes, Pb levels were positively associated with levels of magnesium (Mg), Zn and aluminum (Al), whereas a negative association was observed with the levels of molybdenum (Mo). Furthermore, Cd levels negatively correlated with Mg and Zn levels.^[^ ^31^ ^]^ In addition to OP and DDD, osteomalacia is another hallmark of Itai‐itai disease. Autopsies of inhabitants from a Cd‐polluted area, who died 16 years later, revealed that 82% of the dead inhabitants developed osteomalacia.^[^ ^32^ ^]^

Collectively, extensive epidemiological evidence has revealed the occurrence of Cd‐induced OP. Additionally, Cd has been reported to be involved in the development of OA, DDD, and osteomalacia. However, epidemiological evidence regarding the associations between other heavy metals and degenerative bone diseases is still limited.

### 2.2 Heavy Metal‐Induced Anemia

Epidemiological studies have revealed that Cd, Pb, and Cu were significantly elevated in the sera of children with IDA than in healthy controls.^[^ ^33^ ^]^ The ratios of heavy metals, Cd and Pb, to essential metals, iron and zinc (Zn), were significantly higher in anemic children than in non‐anemic children.^[^ ^34^ ^]^ Furthermore, patients with thalassemia major (TM) exerted higher Cd levels, although at nontoxic levels, but lower levels of Al, Pb, and Zn when compared with the control group.^[^ ^35^ ^]^ In adolescents and women of reproductive age, elevated Cd levels were associated with the prevalence of ID and IDA.^[^ ^36^ ^]^ In deficiencies of some essential metals, including Fe, Cu, Zn, and Ca, uptake of Cd is favorable, leading to anemia.^[^ ^37^ ^]^

As a well‐known heavy metal that induces anemia, Cd causes anemia by inducing ID,^[^ ^38^ ^]^ hemolysis^[^ ^39^ ^]^ and impaired erythropoietin (EPO) production.^[^ ^40^ ^]^ The cytoskeletons of RBCs can be destroyed at the beginning of Cd exposure, resulting in deformed cell membranes.^[^ ^41^ ^]^ In contrast to Cd, Pb induces anemia by inhibiting pyrimidine 5’ nucleotidase activity,^[^ ^42^ ^]^ decreasing the heme amount,^[^ ^43^ ^]^ and inducing erythrophagocytosis and externalization of phosphatidylserine in RBCs.^[^ ^44^ ^]^ Hg can induce nephrotic syndrome^[^ ^45^ ^]^ and aplastic anemia.^[^ ^46^ ^]^ As was demonstrated to impair erythropoiesis and induce anemia by suppressing the formation of burst‐forming unit‐erythroid (BFU‐E) colony even at low doses.^[^ ^47^ ^]^

### 2.3 Anemia as a Risk for Degenerative Bone Diseases

Reportedly, anemia associated with aging can be correlated with an increased risk of low BMD and fractures, particularly in elderly populations.^[^ ^48^, ^49^ ^]^ A cohort study enrolling 371 postmenopausal Turkish women reported that the femur BMD, femur *T*‐score, femur *Z*‐score, spinal BMD, spinal *T*‐score and spinal *Z*‐score were significantly lower in anemic patients than in non‐anemic patients. Furthermore, anemia was identified as an independent predictor of low bone mass in the spine of these patients.^[^ ^50^ ^]^ Another study that recruited 35751 patients with IDA and 178755 individuals without IDA in Taiwan identified that prior IDA was a significant and independent risk factor for developing OP. Furthermore, the risk of OP in patients with IDA increased following intravenous iron therapy but decreased with blood transfusion.^[^ ^51^ ^]^ A population‐based retrospective cohort study also revealed that residents diagnosed with pernicious anemia, a type of anemia attributed to vitamin B12 deficiency, demonstrated a significant increase in proximal femur fractures, vertebral fractures and distal forearm fractures.^[^ ^52^ ^]^ Similar results were observed in community‐dwelling older Korean adults with anemia, particularly in men (*n*  =  72131).^[^ ^53^ ^]^

Moreover, it has been well established that patients with thalassemia develop OP. Notably, OP occurs in more than 40% of patients with beta‐thalassemia, particularly in patients with TM over 20 years of age. In the case of alpha‐thalassemia, about 15% of patients developed OP.^[^ ^54^ ^]^ BMD loss and fracture frequency were found to be elevated in men with beta‐thalassemia than in women.^[^ ^54^ ^]^ Additionally, it has been estimated that fractures occurred in 20% of patients with thalassemia‐associated OP.^[^ ^55^ ^]^ In the United States, a cross‐sectional study investigating adult patients with SCD revealed that 72% of patients demonstrated low BMD, particularly in the lumbar spine, 40% developed OP, and 31% developed osteopenia. Furthermore, the prevalence of OP and osteopenia was higher in young adult patients.^[^ ^56^ ^]^ OP and osteopenia were also prevalent in Saudi Arabia patients with SCD.^[^ ^57^ ^]^

Therefore, the occurrence and risk of OP are increased in anemic cohorts and patients with anemia symptoms induced by nutritional limitations or possessing genetic blood disorders. The elderly population is at the highest risk for developing both anemia and OP; more importantly, they are more sensitive to exogenous etiologies, such as heavy metal exposure.

## 3 MOAs and AOPs Underlying Anemia‐Independent Effects of Heavy Metals on Bone

### 3.1 Imbalanced Bone Formation and Resorption

**Figure 2:**
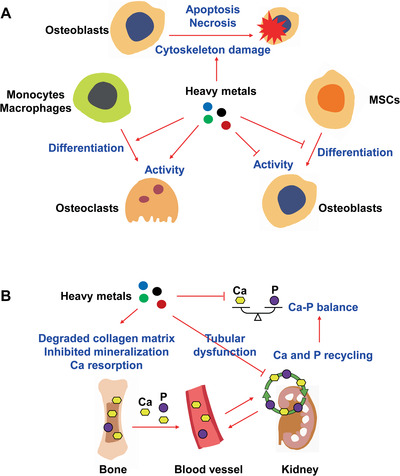
Imbalanced bone formation and resorption, and disturbed homeostasis of essential trace elements in the bone under exposure to heavy metals. A) Under exposure to heavy metals, bone formation is suppressed by inhibiting the activity of osteoblasts and the differentiation of mesenchymal stem cells (MSCs) into osteoblasts. Cytoskeleton damage and cell death of osteoblasts through the induction of apoptosis or necrosis are also involved in the suppression of bone formation. Additionally, bone resorption is enhanced by increasing the activity of osteoblasts and promoting the differentiation of monocytes/macrophages into osteoclasts. B) Degradation of the collagen matrix, inhibition of mineralization and increase of calcium (Ca) resorption are induced by heavy metals, leading to the excretion of Ca and phosphorus (P). Heavy metal‐induced tubular dysfunction inhibits the resorption and recycling of Ca and P in the kidney. These outcomes ultimately induce the loss of Ca and P.

It has been suggested that inhibited bone formation and enhanced bone resorption, mediated by manipulating the differentiation and activities of osteoblasts and osteoclasts, are the primary mechanisms underlying Cd‐induced bone damage (**Figure** **2**A). Using deoxypyridinoline (DPD) as a marker of bone resorption, it was found that urinary Cd levels positively correlated with high DPD secretion, even when exposed to Cd at an early age and relatively low environmental levels. Additionally, clinical consequences were observed in adult life.^[^ ^58^ ^]^ Dietary uptake of Cd was related to the risk of renal tubular dysfunction and accelerated bone resorption, indicating imbalanced bone metabolism under Cd exposure.^[^ ^59^ ^]^Reportedly, Cr reduced the survival and activity of osteoblast‐like cells at various concentrations.^[^ ^60^ ^]^

Following intraperitoneal injection of Cd, the elevated bone resorption decreased the organic and mineral bone contents.^[^ ^61^ ^]^ Compared with untreated rats, decreased BMD, trabecular thickness, increased intertrabecular space, and increased osteoclast activity were observed in rats treated with Cd.^[^ ^62^, ^63^ ^]^ Even at low environmental concentrations, Cd impaired bone formation but enhanced bone resorption in bone cell and organ culture systems.^[^ ^64^ ^]^ In human osteoblast‐like cells, enhanced cell apoptosis and disrupted cytoskeletons were observed upon Cd exposure.^[^ ^65^ ^]^ An in vivo study demonstrated that Cd suppressed bone formation and promoted bone resorption even at moderate concentrations.^[^ ^66^ ^]^ In Cd‐treated mice, the histological bone structure was altered, and osteoclasts were increased as revealed by the upregulated expression of osteoclast marker proteins in the bone marrow, including tartrate‐resistant acid phosphatase (TRAP), matrix metalloproteinase‐9 (MMP9), cathepsin K and carbonic anhydrases.^[^ ^67^ ^]^

### 3.2 Disturbed Homeostasis of Essential Trace Elements in Bone

In additional to imbalanced bone formation and resorption, disordered homeostasis of essential trace elements in the bone is another primary mechanism underlying bone damage under exposure to heavy metals (Figure 2B). After long‐term Cd exposure at 5 and 25 mg L^−1^, levels of Fe, Mn and Zn were significantly increased, while Ca and P levels were decreased in the bone of treated mice.^[^ ^67^ ^]^ Tubular dysfunction as indicated by the increased level of urinary *β*_2_‐microglobulin was observed in women upon long‐term Cd exposure.^[^ ^68^ ^]^ In Cd‐exposed women, low BMD was associated with higher urinary *β*_2_‐microglobulin levels.^[^ ^69^ ^]^ Thus, tubular dysfunction could play a fundamental role in inducing an imbalance between Ca and phosphorous (P) following Cd exposure.^[^ ^70^ ^]^ In contrast, tubular dysfunction was not involved in Cd‐induced damage in the skeleton of men, although urinary Cd levels negatively correlated with BMD.^[^ ^71^ ^]^ The severity of osteomalacia in patients with Itai‐itai disease was found to correlate with the damage in proximal tubules, reduced serum Ca, serum Ca × P, increased urinary P2‐microglobulin and reduced tubular reabsorption of phosphate. The impaired mitochondria in the degenerating proximal tubules accounted for disturbed Ca and P homeostasis resulting in osteomalacia.^[^ ^32^ ^]^ Furthermore, an in vitro study demonstrated the degradation of the collagen matrix and inhibition of mineralization after Cd exposure at toxic concentrations.^[^ ^66^ ^]^

### 3.3 AOPs Underlying Heavy Metal‐Induced Damage in Bone

**Figure 3:**
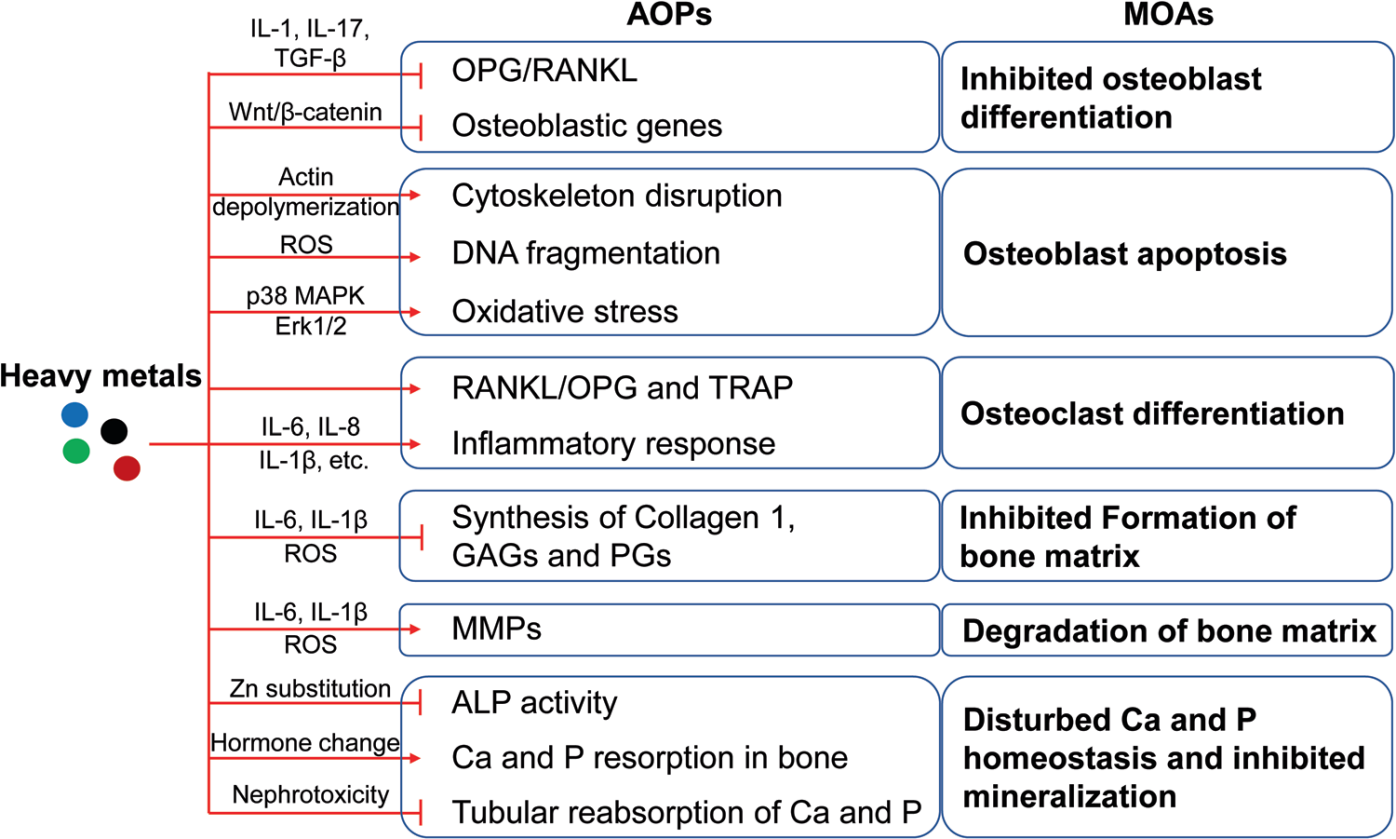
MOAs and AOPs underlying anemia‐independent detrimental effects on bone upon heavy metal exposure. Dysregulated osteogenesis and osteoclastogenesis are largely induced by alterations in the OPG/RANKL ratio and the altered expression of osteoblastic and osteoclastic genes. Oxidative stress and/or inflammation not only alter the expression of osteoblastic and osteoclastic genes, but also induce direct effects on bone. Additionally, oxidative stress and/or inflammation are actively involved in inhibiting the formation and enhancing the degradation of the extracellular bone matrix. Suppressed ALP activity and disturbed Ca and P homeostasis contribute to inhibited bone mineralization. MOAs, modes of action; AOPs, adverse outcome pathways; OPG, osteoprotegerin; RANKL, receptor activator of NF‐*κ*B ligand; TRAP, tartrate‐resistant acid phosphatase; MMPs, matrix metalloproteinases; GAGs, glycosaminoglycans; PGs, proteoglycans; ALP, alkaline phosphatase.

In general, two mechanisms have been proposed for the anemia‐independent detrimental effects of heavy metals on bone metabolism (**Figure** **3**). Cd can directly reduce the expression of genes required for osteoblastic differentiation, extracellular bone matrix formation and mineralization processes. At low doses, long‐term Cd exposure reduced the ratio of OPG and receptor activator of NF‐*κ*B ligand (RANKL), and decreased the expression of key genes (OSX, OPN, COL1A2, and RUNX2) during early osteogenic differentiation of rat mesenchymal stem cells (MSCs), indicating inhibited osteogenesis of MSCs. Although a renal Cd load was present, no increase in urinary Ca excretion and no obvious renal histological changes were observed. These findings suggested that chronic Cd exposure at low doses could directly act on MSCs by altering the RANKL/OPG pathway and inhibiting the expression of the key genes involved in the osteogenic differentiation of MSCs.^[^ ^72^ ^]^ Furthermore, chronic low‐dose exposure to Cd decreased subchondral bone volume and increased the percentage of yellow bone marrow in the tibia of rats, indicating inhibited differentiation of MSCs to osteoblasts but favored differentiation into adipocytes.^[^ ^73^ ^]^ Moreover, Cd inhibited osteogenic differentiation of bone marrow MSCs (BMMSCs) by downregulating osteoblast‐related genes (RUNX2, OCN, OSX, and OPN) and suppressing alkaline phosphatase (ALP), via inhibition on the Wnt/*β*‐catenin pathway, as demonstrated by the reduced protein levels of *β*‐catenin, Wnt3a lymphoid enhancer factor 1 (LEF1) and T‐cell factor 1 (TCF1).^[^ ^74^ ^]^ Additionally, Cd exposure induced osteoblast apoptosis by cytoskeleton disruption (via actin depolymerization),^[^ ^75^ ^]^ DNA fragmentation (by inducing reactive oxygen species (ROS))^[^ ^76^ ^]^ and oxidative stress (by activating the p38 MAPK pathway and inhibiting the Erk1/2 pathway).^[^ ^77^ ^]^

In the meanwhile, Cd promoted bone resorption, as indicated by the increased TRAP‐positive osteoclasts. Mechanistically, Cd stimulated osteoclastogenesis by inducing the expression of RANKL and TRAP activity. Furthermore, in the bone marrow cells and bone tissue, RANKL expression was increased by Cd, whereas OPG expression was decreased, suggesting the important role of the dysregulated OPG/RANKL pathway in Cd‐induced bone damage.^[^ ^67^ ^]^ Treatment with Co and nickel (Ni) inhibited collagen 1 and OPG synthesis, but increased the secretion of RANKL, IL‐6 and IL‐8 in osteoblasts.^[^ ^78^ ^]^ The activity of ALP was decreased by Cd in both the cortical and trabecular bones, whereas Ca excretion in the serum and urine was significantly increased.^[^ ^79^ ^]^ Interestingly, chronic Cd exposure inhibited both serum and hepatic ALP activity in rats.^[^ ^80^ ^]^

Furthermore, acute exposure to Cd promoted the expression of enzymes related to the degradation of the extracellular matrix, including MMP1, MMP3, MMP9, and MMP13. The expression of aggrecan and Col‐II was affected, and levels of glycosaminoglycans (GAGs) and proteoglycans (PGs) decreased. These Cd‐induced changes were mediated by an inflammatory response (IL‐1*β* and IL‐6) and ROS production (OH^•−^ and H_2_O_2_).^[^ ^81^ ^]^ Cd exposure induced nephrotoxicity through impaired vitamin D metabolism in the kidney, leading to subsequent impairment of bone metabolism and bone fragility.^[^ ^82^ ^]^

### 3.4 Oxidative Stress‐Mediated Damage in Bone Induced by Heavy Metals

Oxidative stress has been indicated to play an crucial role in the development of degenerative bone diseases (Figure 3).^[^ ^83^ ^]^ Cd is a well‐known inducer of oxidative stress via inhibition of antioxidant enzymatic and non‐enzymatic activities of thiol group‐rich compounds, such as metallothionein (MT), glutathione (GSH), and vitamins C and E.^[^ ^84^ ^]^ Cd uptake via diet elevated the levels of H_2_O_2_ and decreased the levels of catalase (CAT) and glutathione peroxidase (GPx), which are partly involved in the damaged bone.^[^ ^85^ ^]^ Cd was shown to promote ROS generation and lipoperoxidation by depleting GSH in an osteoblastic cell model, Saos‐2.^[^ ^86^ ^]^ The oxidative stress induced by Cd exposure can impair osteogenesis by inhibiting the expression of RUNX2 and regulating the expression of bone matrix proteins. Osteoclasts can boost bone resorption by generating excess ROS.

Most recently, casticin, a flavonoid with anti‐inflammatory and antitumor effects, was reported to significantly reduce cartilage destruction and OARSI grades by markedly inhibiting oxidative stress, inflammation and MMP13 in the cartilage of OA mice. Mechanistically, casticin attenuated arthritis‐related cartilage degeneration through inhibition of the ROS‐mediated NF‐*κ*B signaling pathway both in vitro and in vivo.^[^ ^87^ ^]^ An in vitro assay of avenanthramides (AVAs), exerting antioxidant properties, revealed that AVAs regulated the function of osteocytes and osteoblasts, and prevented the apoptosis of these cells. By contrast, AVAs increased osteoclast apoptosis. Following AVA treatment, upregulated OPG in osteoblasts and down‐regulated RANKL in osteocytes were observed. All these regulations were independent of the activation of nuclear factor erythroid 2‐related factor 2 (Nrf2).^[^ ^88^ ^]^ In patients with DDD, cartilage end plate (CEP) cells exhibited more cell senescence than cells obtained from age‐matched patients with lumbar vertebral fracture (LVF). Moreover, it was observed that SIRT1 could alleviate oxidative stress‐induced senescence of CEP cells by inhibiting the p53/p21 pathway.^[^ ^89^ ^]^ Sparstolonin B was shown to be capable of preventing lumbar intervertebral disc degeneration (IVDD)‑induced oxidative stress, inflammation and apoptosis through NADPH oxidase activation, TLR4/MyD88/NF‑*κ*B and the phosphoinositide 3‑kinase/protein kinase B signaling pathway.^[^ ^90^ ^]^ Forkhead box O1 (FoxO1) plays a pivotal role in preventing physiological oxidative damage in the bone. Following estrogen deficiency, tumor necrosis factor‐alpha (TNF‐*α*) was found to inhibit FoxO1. Increased ROS activated NF‐*κ*B pathway with TNF‐*α* to form a feed‐forward loop to persistently inhibit the FoxO1 protein in BMMSCs, leading to the development of OP.^[^ ^91^ ^]^

## 4 MOAs and AOPs Underlying Anemia‐Related Effects of Heavy Metals on Bone

### 4.1 Remodeling of Bone Microarchitecture Driven by Stress Erythropoiesis

It has been well‐established that heavy metal exposure causes stress erythropoiesis by inducing cell death and inhibiting the differentiation of erythroblasts in bone marrow, inducing hemolysis, and suppressing EPO production.^[^ ^92^, ^93^, ^94^, ^95^ ^]^ Although the underlying mechanisms remain unclear, it has been widely speculated that impaired bone homeostasis can be attributed to the remodeling of the bone marrow microenvironment and hematopoietic niches driven by stress erythropoiesis. In patients with thalassemia intermedia (TI), expanded medullary cavities and pressure on cortical bone resulted in bone loss, pain and skeletal abnormalities.^[^ ^96^ ^]^ These symptoms could be largely induced by enhanced erythropoiesis and pronounced bone marrow expansion in patients with TI.^[^ ^97^ ^]^ Furthermore, treatment with zoledronic acid improved BMD, but failed to attenuate erythropoiesis in patients with TI, suggesting a major role of enhanced erythropoiesis in the observed bone loss.^[^ ^96^ ^]^ For OP in patients with TM, the pathogenic mechanisms are more complicated than those in patients with IT. It was suggested that the OP in patients with TM involved an iron overload, effect of iron chelation therapy, disordered endocrine system, expansion of bone marrow and generic alterations.^[^ ^98^ ^]^ Furthermore, the pronounced activity of osteoclasts contributed to bone destruction in thalassemia patients.^[^ ^99^ ^]^

### 4.2 Suppressed EPO Production as a MOA Underlying Heavy Metal‐Induced Bone Damage

Notably, Cd exposure suppresses EPO production by inducing kidney injury. In patients with Cd‐induced Itai‐itai disease, anemia is closely associated with the progression of renal dysfunction and low serum EPO levels were reported in all the patients, independent anemia severity.^[^ ^100^ ^]^ In rats chronically administrated with Cd, renal tubular damage and hypoproduction of EPO mRNA were observed in the kidneys, resulting in low plasma EPO levels.^[^ ^101^ ^]^ Inhibition of EPO by Pb was observed both in humans and rats, contributing to Pb‐induced anemia.^[^ ^102^ ^]^ Additionally, Cd suppressed renal EPO production through multiple mechanisms, including a direct effect, iron accumulation and destroyed EPO‐producing cells.^[^ ^95^ ^]^

**Figure 4:**
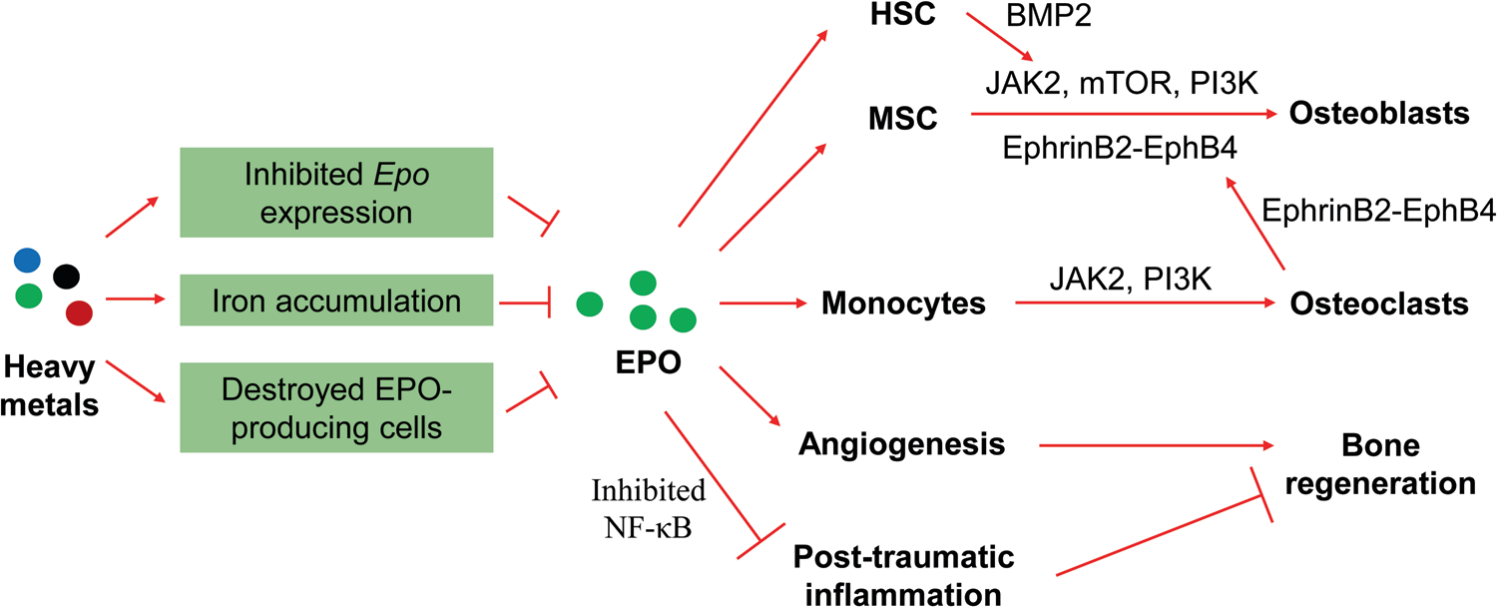
Roles of hypo‐produced EPO in the anemia‐related detrimental effects on the bone under heavy metal exposure. The renal hypoproduction of EPO is induced by heavy metals. EPO deficiency impairs osteogenesis by downregulating several key signaling pathways in osteoblasts and/or osteoblasts/HSCs. Upon EPO deficiency, post‐traumatic inflammation is enhanced, and angiogenesis is impaired during bone healing. Additionally, EPO deficiency fails to induce vascular endothelial growth factor (VEGF) expression and increase peripheral endothelial progenitor cells. EPO, erythropoietin; HSC, hematopoietic stem cell; MSC, mesenchymal stem cell; JAK2, Janus kinase 2; mTOR, mechanistic target of rapamycin; PI3K, phosphoinositide 3‐kinase; BMP2, bone morphogenetic protein 2.

In general, the role of EPO in regulating bone metabolism is that of a double‐edged sword (**Figure** **4**). It is widely accepted that EPO stimulates osteoclastogenesis; however, the effects of EPO on osteogenesis remain controversial owing to the doses used. In vitro studies reported that EPO (10–100 U mL^−1^) exerted stimulatory effects on osteogenic differentiation and mineralization.^[^ ^103^, ^104^, ^105^ ^]^ Janus kinase 2 (JAK2), mechanistic target of rapamycin (mTOR), phosphoinositide 3‐kinase (PI3K) and EphrinB2‐EphB4 signaling pathways were reportedly involved in EPO‐stimulated osteogenesis in vitro.^[^ ^103^, ^104^, ^105^ ^]^ However, EPO treatment at 1–10 U mL^−1^, closer to the clinical EPO range, was shown to demonstrate no effect on osteogenesis.^[^ ^104^, ^106^ ^]^ In contrast, EPO treatment at 5 to 20 U mL^−1^ promoted in vitro osteoclast generation,^[^ ^103^, ^105^, ^106^ ^]^ dependent on JAK2 and PI3K signaling pathways and independent of MAPK signaling pathway.^[^ ^106^ ^]^

In addition to direct effects on osteogenesis, EPO conducted indirect effects on osteogenesis by regulating hematopoietic stem cells (HSCs) and osteoclasts. EPO increased the expression of bone morphogenetic protein 2 (BMP2) in HSCs by promoting the HSC population and in turn, BMP2 stimulated differentiation of osteoblasts.^[^ ^107^ ^]^ The EphrinB2‐EphB4 signaling pathway is a bidirectional signaling pathway that regulates osteogenesis, where EphrinB2 from osteoclasts binds to its receptor, EphB4, on osteoblasts.^[^ ^108^ ^]^ EPO was reported to increase the expression of EphrinB2 in osteoclasts and that of EphB4 in both osteoblastic cells and bone marrow stromal cells (osteoblast progenitors).^[^ ^105^ ^]^ Furthermore, EPO‐inhibited post‐traumatic inflammatory response and EPO‐promoted angiogenesis, a crucial process for bone healing, are involved in the beneficial effects of EPO on bone regeneration. Low dose long‐term EPO treatment stimulated bone healing in mice by inhibiting the NF‐*κ*B signaling pathway.^[^ ^109^ ^]^ Furthermore, EPO promoted angiogenesis by increasing the expression of vascular endothelial growth factor (VEGF)^[^ ^110^ ^]^ and the number of peripheral endothelial progenitor cells.^[^ ^109^ ^]^ However, EPO‐promoted angiogenesis was probably dependent of osteoclastogenesis.^[^ ^111^ ^]^

### 4.3 Impact of Disordered Iron Homeostasis on Erythropoiesis and Bone Homeostasis

IDA is the most frequent reported anemia.^[^ ^18^ ^]^ In the human body, 70% of the total iron is incorporated into heme, the core of hemoglobin.^[^ ^112^ ^]^ The HRI‐eIF2*α*P‐ATF4 signaling pathway is the master regulator of erythropoiesis under ID condition.^[^ ^113^, ^114^ ^]^ Heme‐regulated eIF2*α* kinase, also known as the heme‐regulated inhibitor (HRI), is activated in erythroblasts to maintain stress erythropoiesis under various stress conditions, such as ID, oxidative stress and environmental stress.^[^ ^113^, ^114^ ^]^ Thus, long‐term ID causes hypochromic microcytic anemia, characterized by decreased RBC counts and hemoglobin levels, as well as paler RBCs reduced in size.^[^ ^113^, ^114^ ^]^ More importantly, our previous study revealed that the HRI was required for the survival and differentiation of erythroid progenitor cells following Cd treatment.^[^ ^92^ ^]^ Additionally, we observed that HRI played a crucial role in protecting the liver and kidney against Cd‐induced injuries.^[^ ^115^ ^]^ Upon Pb exposure, the HRI was required to prevent hemolysis and inhibition of erythroid differentiation. Furthermore, HRI increased splenic iron availability for extramedullary erythropoiesis by promoting hepatic hepcidin expression upon Pb exposure.^[^ ^94^ ^]^

**Figure 5:**
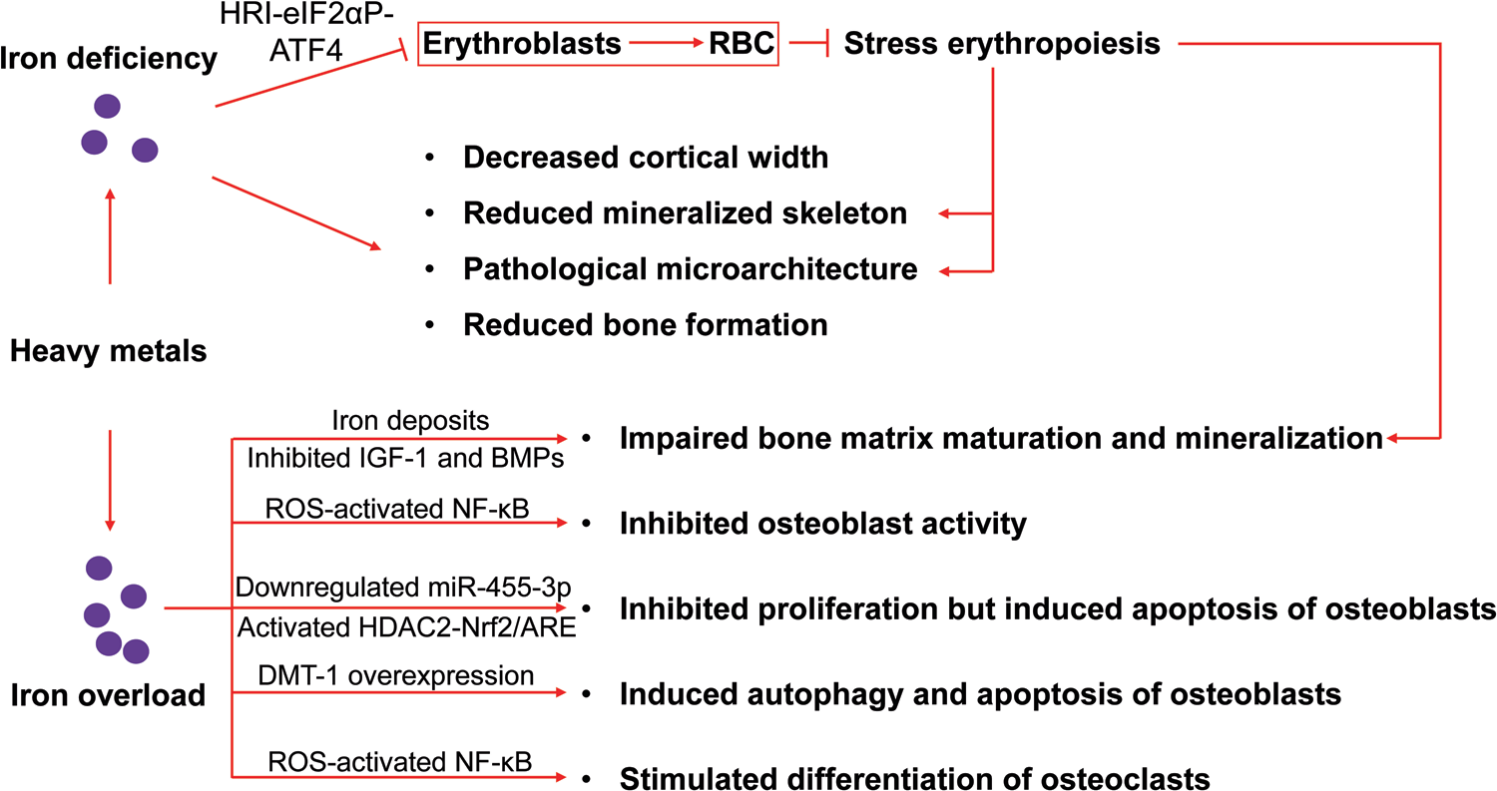
Mechanisms underlying the bone damage mediated by iron deficiency and iron overload. Both iron deficiency and iron overload can cause detrimental effects on bone metabolism upon heavy metal exposure. The HRI‐eIF2αP‐ATF4 signaling pathway might be involved in bone damage under iron deficiency via regulation of stress erythropoiesis. Dysregulated hepcidin, BMP, and IGF signaling pathways could be involved in bone damage observed under iron overload. The induction of oxidative stress, dysregulated microRNA and histone deacetylation by iron overload could contribute to dysregulated osteogenesis and osteoblastogenesis. RBC, red blood cell; ROS, reactive oxygen species; DMT‐1, divalent metal transporter 1; IGF‐1, insulin‐like growth factor 1; BMPs, bone morphogenetic proteins.

It was observed that rats with severe ID demonstrated decreased cortical width of the femur and tibia, reduced mineralized skeleton, and pathological modulation of microarchitecture in the vertebral trabecular bone.^[^ ^116^ ^]^ Additionally, ID was associated with declining biochemical markers of bone formation.^[^ ^117^ ^]^ After adjusting for BMI and age, the ID status in premenopausal women significantly increased levels of a bone resorption marker (NTx) and marginally decreased a bone formation marker (P1NP).^[^ ^118^ ^]^ These observations indicated that in premenopausal women with ID, bone resorption goes beyond bone formation (**Figure** **5**).

Ironically, iron overload has also been implicated in the development of degenerative bone diseases (Figure 5). In some patients with hemochromatosis, iron overload resulted in osteopenia.^[^ ^119^ ^]^ In patients with suboptimally treated TM, iron deposited along the osteoid surfaces and mineralization fronts, and focal thickened osteoid seams appeared together with focal iron deposits. These observations were obtained in both child and adolescent patients.^[^ ^120^ ^]^ Moreover, iron deposits in bone and low circulating insulin‐like growth factor 1 (IGF‐1) levels could partly contribute to the pathogenesis of low BMD, impaired bone matrix maturation and defective mineralization in these patients.^[^ ^120^ ^]^

Hepcidin1 knockout mice were found to develop low bone mass and altered bone microarchitecture probably owing to excessive iron‐induced decrease in osteoblastic activity. In hepcidin1 knockout mice, impaired bone formation induced by iron overload was probably caused by altered BMP signaling pathway.^[^ ^121^ ^]^ Excess iron‐induced oxidative stress inhibited the activity of osteoblasts,^[^ ^122^ ^]^ but stimulated the differentiation of osteoclasts, by activating the NF‐*κ*B signaling pathway.^[^ ^123^ ^]^ Ferric ammonium citrate (FAC), a donor of ferric ions, inhibited osteoblast proliferation and promoted osteoblast apoptosis by downregulating miR‐455‐3p and upregulating HDAC2. Overexpression of miR‐455‐3p eliminated these detrimental effects of FAC on osteoblasts. Additionally, miR‐455‐3p overexpression inhibited oxidative stress‐induced injury in mice with OP. It was further demonstrated that miR‐455‐3p functioned by regulating HDAC2‐Nrf2/ARE signaling pathway.^[^ ^124^ ^]^ Moreover, iron overload ameliorated the functions of primary osteoblasts in mice by increasing ROS, while iron depletion could reverse this adverse effect by inhibiting the functions of primary osteoclasts. Hepcidin overexpression attenuated OP processes in mice, implying that hepcidin could be a prospective drug target for preventing postmenopausal osteoporosis.^[^ ^125^ ^]^ Furthermore, the overexpression of divalent metal transporter 1 (DMT‐1) in osteoblasts induced autophagy and apoptosis in osteoblasts, thus promoting OP, by inducing cellular iron overload.^[^ ^126^ ^]^

### 4.4 Detrimental Effects of Heavy Metals on Iron Homeostasis

Although Cd exposure can increase the expression of DMT‐1 in enterocytes, Cd competes with iron uptake in the intestine through binding to DMT‐1.^[^ ^127^ ^]^ Our recent study demonstrated reduced expression of ferroportin (FPN), the only known iron exporter, and the decreased cellular labile iron pool (LIP) in macrophages upon Cd exposure.^[^ ^128^ ^]^ We further showed that Cd regulated the expression of FPN by competing with iron to bind to the 5′‐UTR iron‐responsive element (IRE) within the FPN’ mRNA.^[^ ^128^ ^]^ Additionally, Cd caused hepcidin‐mediated ID by inducing interleukin 6 (IL‐6)^[^ ^129^ ^]^ and suppressing the synthesis of EPO in the kidney.^[^ ^41^ ^]^ Intriguingly, it was reported that ID promoted the uptake and renal accumulation of Cd via the dietary exposure route.^[^ ^130^ ^]^

Furthermore, elevated serum Pb levels inhibited iron uptake and thus induce anemia in children.^[^ ^131^ ^]^ Iron accumulation in the kidney was observed following long‐term Pb exposure.^[^ ^132^ ^]^ Additionally, porphyria was induced by Pb exposure.^[^ ^133^ ^]^ Hg was found to be positively associated with indicators of serum iron status, such as ferritin.^[^ ^134^ ^]^ The ratio of serum ferritin to soluble transferrin receptor in serum (sTfR/S‐Ft) was negatively associated with the Hg levels.^[^ ^134^ ^]^ However, serum Hg levels was found to be inversely correlated with serum ferritin levels,^[^ ^135^ ^]^ while no relationship was observed between them in vitro.^[^ ^136^ ^]^ Inactivation of pyruvate dehydrogenase by As was suggested to disrupt iron homeostasis.^[^ ^137^ ^]^ As‐induced nitric oxide (NO)^[^ ^138^ ^]^ can interact with iron‐responsive element‐binding proteins (IRPs) to regulate the expression of IRE‐containing mRNAs encoding ferritin and transferrin, resulting in altered cellular LIP.^[^ ^139^ ^]^ Three Cu oxidases, ceruloplasmin, hephaestin, and zyklopen, have also been reported to be involved in regulating iron homeostasis.^[^ ^140^ ^]^

Taken together, heavy metals disturb iron homeostasis and iron‐related regulation through several mechanisms, including binding to iron transporters, interrupting iron‐sensing and iron‐regulated pathways, competing with iron and generating free radicals. Compared with ID‐induced anemia, both ID and iron overload are detrimental to bone homeostasis, leading to an increased risk of developing degenerative bone diseases.

## 5 The Integrated Contributions and Cross‐Talks among Intertwined Mechanisms Responsible for Heavy Metal‐Induced Havocs on Bone

**Figure 6:**
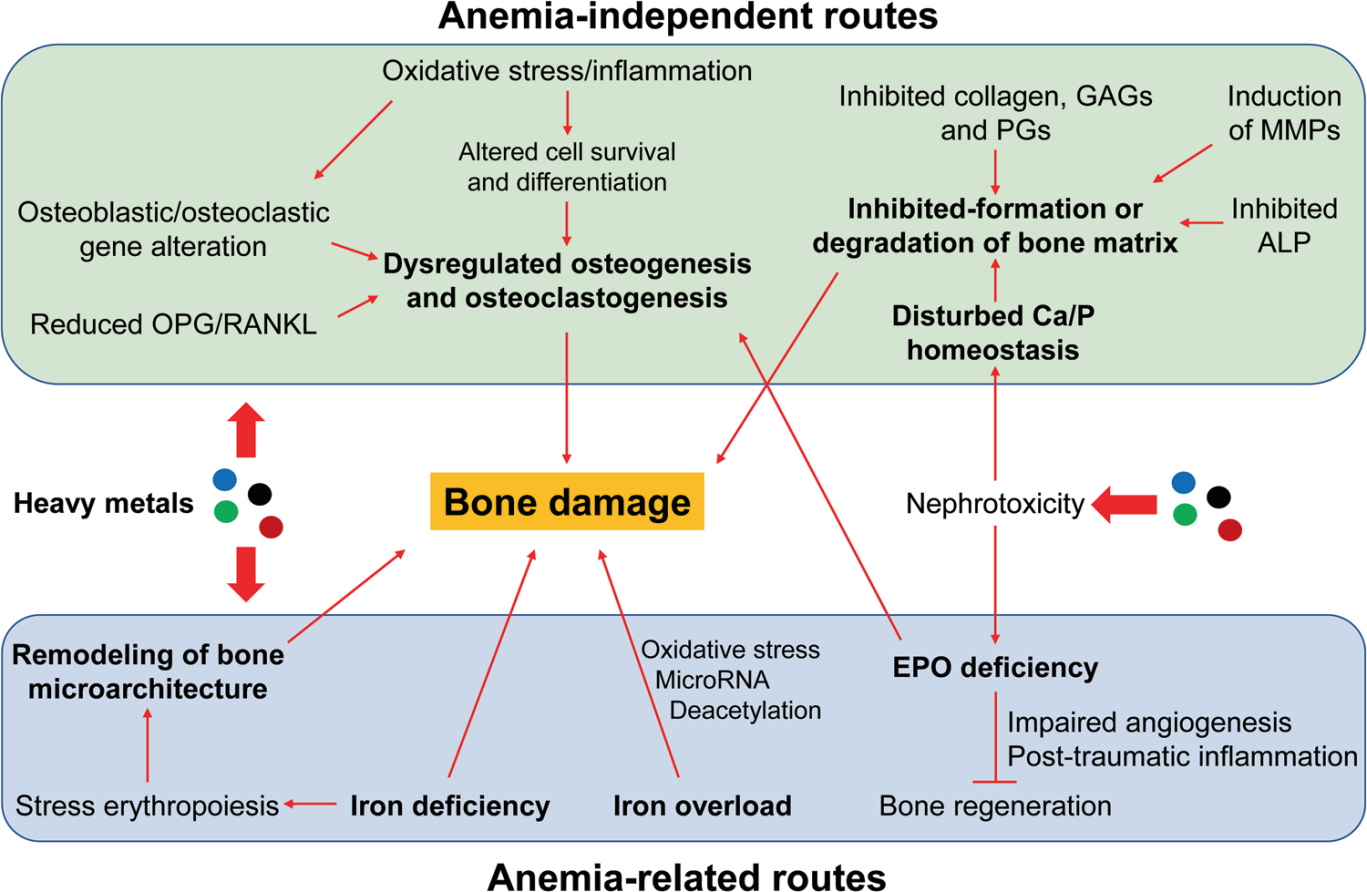
A global view of MOAs and AOPs underlying heavy metal‐induced bone damage. Various modes of action (MOAs) and adverse outcome pathways (AOPs) are involved in heavy metal‐induced bone damage through anemia‐independent and ‐related routes. The MOAs involved in the anemia‐independent routes mainly include dysregulated osteogenesis and osteoblastogenesis, inhibited formation and enhanced degradation of the extracellular bone matrix, disturbed Ca/P homeostasis, and inhibited mineralization. Altered OPG/RANKL ratio and altered expression of osteoblastic and osteoclastic genes are key AOPs underlying dysregulated osteogenesis and osteoblastogenesis. Nephrotoxicity is the main mechanism for the disordered Ca/P homeostasis, contributing to damage in the bone matrix together with altered levels of other matrix essentials and ALP. Regarding anemia‐related routes, stress erythropoiesis‐driven remodeling of bone microarchitecture, renal EPO hypoproduction, and disordered iron homeostasis are the main MOAs. Nephrotoxicity induces the hypoproduction of EPO, which further impairs bone generation and contributes to dysregulated osteogenesis and osteoblastogenesis. Remodeling of bone microarchitecture induces physical damage to the bone, while ID and iron overload cause physicochemical damage on bone. Importantly, oxidative stress contributes to both anemia‐independent and ‐related bone damage upon heavy metal exposure. OPG, osteoprotegerin; RANKL, receptor activator of NF‐*κ*B ligand; EPO, erythropoietin; GAGs, glycosaminoglycans; PGs, proteoglycans; MMPs, matrix metalloproteinases; ALP, alkaline phosphatase; ID, iron deficiency.

As summarized in **Table** **1** and shown in **Figure** **6**, both anemia‐independent and ‐related detrimental effects of heavy metals on bone are mediated by multiple MOAs and AOPs. Regarding anemia‐independent bone damage induced by heavy metals, dysregulated osteogenesis and osteoclastogenesis are the major MOAs, which partially trigger other MOAs (e.g., inhibited formation and enhanced degradation of the extracellular bone matrix as well as inhibited mineralization). Reduced OPG/RANKL ratio and altered expression of osteoblastic and osteoclastic genes largely account for dysregulated osteogenesis and osteoclastogenesis. Heavy metal‐induced oxidative stress and/or inflammation are involved in the changed expression of these osteoblastic and osteoclastic genes via modulation of certain signaling pathways (e.g., Wnt/*β*‐catenin). However, oxidative stress and/or inflammation additionally induce direct effects on the bone, such as osteoblast apoptosis, by inducing damage in the cytoskeleton and DNA, and osteoclast differentiation. Moreover, oxidative stress and/or inflammation are actively involved in inhibiting the formation and enhancing the degradation of the extracellular bone matrix via inhibition of the synthesis of collagen 1, GAGs, and PGs, and the induction of MMP synthesis. The suppression of ALP activity and increase of Ca loss in the bone contribute to the inhibited bone mineralization upon heavy metal exposure. Furthermore, inhibited bone mineralization can be attributed to disturbed Ca and P homeostasis, mainly caused by heavy metal‐induced tubular dysfunction.

**Table 1:** MOAs and AOPs underlying heavy metal‐induced detrimental effects on bone. MOAs, modes of action; AOPs, adverse outcome pathways; Ca, calcium; P, phosphorus; OPG, osteoprotegerin; RANKL, receptor activator of NF‐*κ*B ligand; TRAP, tartrate‐resistant acid phosphatase; MMPs, matrix metalloproteinases; GAGs, glycosaminoglycans; PGs, proteoglycans; ALP, alkaline phosphatase; EPO, erythropoietin; HSC, hematopoietic stem cell; MSC, mesenchymal stem cell; RBC, red blood cell; JAK2, Janus kinase 2; PI3K, phosphoinositide 3‐kinase; mTOR, mechanistic target of rapamycin; BMPs, bone morphogenetic proteins; BMP2, bone morphogenetic protein 2; TGF‐*β*, transforming growth factor *β*; ROS, reactive oxygen species; DMT‐1, divalent metal transporter 1; IGF‐1, insulin‐like growth factor 1; VEGF, vascular endothelial growth factor; IL‐1, interleukin 1; IL‐1*β*, interleukin 1*β*; IL‐6, interleukin 6; IL‐17, interleukin 17; TLR4, toll‐like receptor 4; MAPK, mitogen‐activated protein kinase; Erk1/2, extracellular signal‐regulated kinase 1/2

Routes	MOAs	AOPs	Refs
Anemia‐independent routes	Inhibited osteoblast differentiation	Reduced ratio of OPG and RANKL via altered expression of IL‐1, IL‐17 and TGF‐*β*	^[^ ^72^ ^]^
		Inhibited expression of RUNX2, OSX, OPN, OCN and COL1A2 by suppressing the Wnt/*β*‐catenin pathway	^[^ ^72^, ^74^ ^]^
	Induction of osteoblast apoptosis	Cytoskeleton disruption owing to actin depolymerization	^[^ ^75^ ^]^
		DNA fragmentation probably by inducing ROS	^[^ ^76^ ^]^
		Oxidative stress via activation of p38 MAPK pathway and inhibition of Erk1/2 pathway	^[^ ^77^ ^]^
	Enhanced osteoclast differentiation	Increased ratio of RANKL/OPG and TRAP activity	^[^ ^67^, ^78^ ^]^
		Increased levels of IL‐6 and IL‐8 in osteoblasts via TLR4 activation	^[^ ^78^ ^]^
	Inhibited formation and enhanced degradation of extracellular bone matrix	Induction of MMP1, MMP3, MMP9 and MMP13, and decreased synthesis of collagen 1, GAGs and PGs via inflammatory response (IL‐1*β* and IL‐6) and ROS production	^[^ ^78^, ^81^ ^]^
	Disturbed Ca and P homeostasis and inhibited mineralization	Suppressed ALP activity via Zn substitution and increased excretion of Ca in serum and urine	^[^ ^79^, ^80^ ^]^
		Altered hormonal status and tubular dysfunction induce: enhanced Ca and P resorption in bone, decreased serum Ca and serum Ca × P, reduced tubular reabsorption of Ca and P	^[^ ^59^, ^82^ ^]^
Anemia‐related routes	Remodeling of bone microarchitecture driven by stress erythropoiesis	Altered bone mechanical properties probably owing to medullary cavity expansion and the alteration of bone matrix	^[^ ^96^, ^97^ ^]^
	Renal EPO‐hypoproduction	Suppression of EPO expression, iron accumulation and destroyed EPO‐producing cells	^[^ ^95^ ^]^
	Impaired osteogenic differentiation due to EPO deficiency	Impaired JAK2, mTOR, PI3K and EphrinB2‐EphB4 signaling pathways in osteoblasts	^[^ ^103^, ^104^, ^105^ ^]^
		Impaired support from the neighboring cells: BMP2 signaling pathway by HSCs, EphrinB2‐EphB4 signaling pathway by osteoclasts	^[^ ^107^, ^108^ ^]^
	EPO‐promoted osteoclastogenenesis	Enhanced JAK2 and PI3K signaling pathways	^[^ ^103^, ^105^, ^106^ ^]^
	Impaired bone regeneration due to EPO deficiency	Promoted post‐traumatic inflammation and impaired angiogenesis owing to a lack of EPO‐inhibited NF‐*κ*B signaling pathway, EPO‐promoted VEGF expression and peripheral endothelial progenitor cells	^[^ ^109^, ^110^ ^]^
	Iron deficiency	Stress erythropoiesis regulated by HRI‐eIF2*α*P‐ATF4 signaling pathway	^[^ ^92^, ^94^, ^113^, ^114^, ^115^ ^]^
		Decreased cortical width of femur and tibia, reduced mineralized skeleton, pathological modulation of microarchitecture in the vertebral trabecular bone, and reduced bone formation	^[^ ^116^, ^117^ ^]^
	Iron overload	Iron deposits in bone and low circulating IGF‐1 levels, resulting in impaired bone matrix maturation and defective mineralization	^[^ ^120^ ^]^
		Impaired BMP signaling pathway related to hepcidin expression	^[^ ^121^, ^125^ ^]^
		Inhibition of osteoblast activity by ROS‐activated NF‐*κ*B signaling pathway	^[^ ^123^, ^125^ ^]^
		Inhibited osteoblast proliferation, enhanced osteoblast apoptosis and induction of oxidative stress via downregulation of miR‐455‐3p and upregulation of HDAC2‐Nrf2/ARE	^[^ ^124^ ^]^
		DMT‐1 overexpression‐mediated autophagy and apoptosis of osteoblasts	^[^ ^126^ ^]^
		Stimulated differentiation of osteoclasts by ROS‐activated NF‐*κ*B signaling pathway	^[^ ^123^ ^]^

Regarding anemia‐dependent bone damage upon heavy metal exposure, direct evidence at the molecular level is rather limited, even though heavy metals are tightly correlated with anemia‐dependent bone damage. Nonetheless, the involvement of two types of MOAs, physical and biochemical MOAs, has been proposed. The physical MOA involves the remodeling of bone microarchitecture, driven by stress erythropoiesis, such as altered bone mechanical properties owing to medullary cavity expansion and alteration of bone matrix. However, the underlying molecular mechanisms remain largely unknown. On the other hand, heavy metal exposure causes hypoproduction of EPO in the kidney by directly suppressing EPO expression, accumulating iron, and destroying EPO‐producing cells. This significantly impairs multiple EPO‐mediated bone homeostasis and regeneration. EPO deficiency impairs osteogenesis owing to the downregulation of JAK2, mTOR, PI3K, and EphrinB2‐EphB4 signaling pathways in osteoblasts and/or osteoblasts. During bone healing, post‐traumatic inflammation is enhanced and angiogenesis is impaired without the inhibition of the NF‐*κ*B signaling pathway by EPO. Moreover, EPO is necessary for VEGF expression and increasing peripheral endothelial progenitor cells. Thus, EPO deficiency would be an important MOA involved in heavy metal‐induced bone damage. Both ID and iron overload can be induced upon heavy metal exposure, and both can induce detrimental effects on bone metabolism. The HRI‐eIF2aP‐ATF4 signaling pathway mainly regulates stress erythropoiesis and might be involved in the bone damage under iron deficiency. Additionally, dysregulated hepcidin, BMP, and IGF signaling pathways have been implicated in bone damage under iron overload. Iron overload also induces oxidative stress, which could further inhibit osteoblast activity and stimulate osteoclast differentiation by activating the NF‐*κ*B signaling pathway. Moreover, inhibition of osteoblast proliferation and promotion of osteoblast apoptosis could be induced by dysregulated microRNA (e.g., miR‐455‐3p) and histone deacetylation under iron overload, in which oxidative stress also plays a crucial role.

## 6 Conclusions and Perspectives

In the current review, we summarize the occurrence and mechanisms of degenerative bone diseases with or without the involvement of anemia under exposure to heavy metals. We recapitulate the detrimental effects of heavy metals on bone homeostasis, erythropoiesis and iron homeostasis, resulting in degenerative bone diseases and anemia. Extensively epidemiological evidence has proven the increased risks of both degenerative bone diseases and anemia induced by heavy metals. Additionally, anemia‐promoted degenerative bone diseases have been established. An imbalance of bone formation and resorption and disturbed homeostasis of essential trace elements in bone tissue are the predominant MOAs underlying anemia‐independent degenerative bone diseases induced by heavy metals. Remodeling of bone microarchitecture, impaired production of EPO, and disordered iron homeostasis could be the MOAs underlying anemia‐associated degenerative bone diseases under exposure to heavy metals. Furthermore, oxidative stress is a common mechanism involved in both anemia‐dependent and ‐independent routes. Thus, these MOAs and AOPs could bridge heavy metals, degenerative bone diseases, and anemia together, highlighting the role of anemia‐related degenerative bone diseases under exposure to heavy metals. Importantly, the elderly and anemic populations, such as women and patients with genetic blood disorders, are at high risk for heavy metal‐induced degenerative bone diseases, both dependent and independent on anemia.

To date, several knowledge gaps exist regarding heavy metal‐induced degenerative bone diseases. First, a prominent issue is the lack of direct evidence associating anemia with heavy metal‐induced degenerative bone diseases, either from epidemiologic studies or animal studies. Hence, these investigations are warranted in the future. Second, except for Cd, epidemiological investigations regarding associations between other heavy metals and degenerative bone diseases remain limited. Unlike heavy metal‐induced OP, little is known regarding the impact and mechanisms of heavy metals on the development of OA, DDD and osteomalacia. Third, AOPs underlying the remodeling of bone microarchitecture upon heavy metal‐induced stress erythropoiesis are largely unknown. Oxidative stress is involved in most anemia‐independent MOAs, while only involved in iron overload in anemia‐related MOAs. Thus, the role of oxidative stress in other anemia‐related MOAs (e.g., EPO hypoproduction) necessitates further investigations. Moreover, it is necessary to identify additional AOPs underlying both anemia‐independent and ‐related detrimental effects of heavy metals on the bone. Fourth, whether synergistic toxic actions and effects between anemia‐independent and anemia‐dependent bone damage exist upon heavy metal exposure is still unknown. Last, it is important to investigate whether both anemia‐independent and anemia‐dependent routes are indispensable and intertwined in inducing bone damage upon heavy metal exposure. All these gaps need further evaluation in future investigations, particularly in susceptible populations.

## Conflict of Interest

The authors declare no conflict of interest.

## Supporting information

Supporting Information
