# Supplementary material for: Adverse Impact of Heavy Metals on Bone Cells and Bone Metabolism Dependently and Independently through Anemia
Source: Adv Sci (Weinh). 2020 Aug 4;7(19):2000383. doi: 10.1002/advs.202000383 (PMC7539179; doi:10.1002/advs.202000383)
Supplement: Supplementary file 1 — Supporting Information [file ADVS-7-2000383-s001.pdf]

2020 Wiley-VCH GmbH

ADVANCED SCIENCE
Open Access

Supporting Information

for *Adv. Sci.*, DOI: 10.1002/advs.202000383

Adverse Impact of Heavy Metals on Bone Cells and Bone Metabolism Dependently and Independently through Anemia

Shuping Zhang, Li Sun, Jie Zhang, Sijin Liu, Jinxiang Han, and Yajun Liu**

Supplementary Table 1. Sources and exposure routes of heavy metals.

	Sources	Human exposure routes	Refs
Cd	Mining, smelting, fertilizers, pigments, batteries, plastics, pesticides	Occupational exposure, inhalation, contaminated food and drinking water	[1, 2]
Pb	Mining, fossil fuel, pesticides, pigments, cosmetics, fertilizers	Occupational exposure, contaminated food and drinking water, smoking	[1, 2]
As	Mining, smelting, pesticides, fertilizers, medical waste	Contaminated drinking water	[1, 3]
Cr	Smelting, fossil fuel, tannery, sewage, fertilizers, paper mill	Occupational exposure, contaminated water	[1, 2]
Hg	Mining, batteries, agriculture, waste water	Contaminated marine food and water	[1]

Supplementary Table 2. Abbreviation list.

**Abbreviation**	**Full name**
MOAs	Modes of action
AOPs	Adverse outcome pathways
Ca	Calcium
P	Phosphorus
OPG	Osteoprotegerin
RANKL	Receptor activator of NF-κB ligand
TRAP	Tartrate-resistant acid phosphatase
MMPs	Matrix metalloproteinases
GAGs	Glycosaminoglycans
PGs	Proteoglycans
ALP	Alkaline phosphatase
EPO	Erythropoietin
HSC	Hematopoietic stem cell
MSC	Mesenchymal stem cell
JAK2	Janus kinase 2
PI3K	Phosphoinositide 3-kinase
mTOR	Mechanistic target of rapamycin

BMP2	Bone morphogenetic protein 2
RBC	Red blood cell
ROS	Reactive oxygen species
DMT-1	Divalent metal transporter 1
IGF-1	Insulin-like growth factor 1
BMPs	Bone morphogenetic proteins
VEGF	Vascular endothelial growth factor
IL-1	Interleukin 1
IL-1β	Interleukin 1β
IL-6	Interleukin 6
IL-17	Interleukin 17
TGF-β	Transforming growth factor β
TLR4	Toll-like receptor 4
MAPK	Mitogen-activated protein kinase
Erk1/2	Extracellular signal-regulated kinase 1/2

References

1. M. Jaishankar, T. Tseten, N. Anbalagan, B. B. Mathew, K. N. Beeregowda, *Interdiscip Toxicol* 2014, ***7***, 60.

2. S. Martin, W. Griswold, *Environ. Sci. Technol.* 2009, ***15***, 1.

3. A. H. Smith, E. O. Lingas, M. Rahman, *Bull World Health Organ* 2000, ***78***, 1093.
